# Bilirubin Levels in Infancy and Their Associations with Body Weight, Levels of Iron-Related Parameters and Steroid Hormone Levels

**DOI:** 10.3390/metabo14070393

**Published:** 2024-07-19

**Authors:** Charlotte Grosse-Thie, Mandy Vogel, Ronny Baber, Uta Ceglarek, Wieland Kiess

**Affiliations:** 1 LIFE Child, LIFE Leipzig Research Center for Civilization Diseases, Leipzig University, Philipp-Rosenthal-Strasse 27, 04103 Leipzig, Germany; mandy.vogel@medizin.uni-leipzig.de (M.V.); ronny.baber@medizin.uni-leipzig.de (R.B.); uta.ceglarek@medizin.uni-leipzig.de (U.C.); wieland.kiess@medizin.uni-leipzig.de (W.K.); 2 Center for Pediatric Research (CPL), Department of Women and Child Health, Hospital for Children and Adolescents, Leipzig University, Liebigstrasse 20a, 04103 Leipzig, Germany; 3 Institute of Laboratory Medicine, Clinical Chemistry and Molecular Diagnostics (ILM), Leipzig University, Paul-List Str. 13/15, 04103 Leipzig, Germany

**Keywords:** bilirubin, infancy, steroid hormones, ferritin, transferrin, body weight

## Abstract

It is assumed that bilirubin is hormonally regulated and influences weight development by preventing weight gain. However, studies in healthy infants are limited. The present study established reference values for bilirubin and investigated whether bilirubin levels are significantly associated with body weight, levels of ferritin and transferrin as well as steroid hormone levels in a study population of three- and six-month-old healthy infants. Data from a total of 411 study visits from the LIFE Child study (Leipzig, Germany) were analyzed. Associations were examined using linear regression analyses. Besides laboratory parameters, anthropometric data were gathered. We found statistically significant associations between body weight and bilirubin levels. In girls, we observed additional associations between bilirubin levels and both ferritin and transferrin concentrations at three months of age. At six months, steroid hormone levels were significantly associated with concentrations of total and indirect bilirubin, with effects differing by sex. Our study thus confirms associations already reported from animal studies and studies in adult populations. Furthermore, we showed that these associations already exist in the first year of life, are influenced by sex and age and, further, depend on the bilirubin type. Our results provide reference values for bilirubin and assist, therefore, in interpreting bilirubin levels in infancy.

## 1. Introduction

As an essential measure for assessing erythrocyte function, liver function and cholestasis parameters, bilirubin is of immense importance in neonatology as well as in pediatric, adolescent and adult medicine [1,2].

Bilirubin is mainly produced during the degradation of hemoglobin, initiated by heme oxygenase [3]. Therefore, humans reach the highest physiological bilirubin concentrations during the neonatal period due to the degradation of fetal hemoglobin.

While bilirubin was initially known exclusively as a potentially toxic waste product of heme metabolism and primary pigment of bile [4], an increasing number of recent studies attribute antioxidant [5], anti-inflammatory [6] and immunomodulatory [7] effects to bilirubin via the inhibition of NADPH oxidase complexes [8]. Furthermore, animal experiments have shown that bilirubin concentrations are apparently hormonally regulated [9] and may influence body weight [10]. In analyses of a multicenter study, it was reported that term newborns with higher neonatal bilirubin levels had a lower risk of developing obesity during childhood [11]. In addition, adults with Gilbert’s syndrome, who typically have elevated indirect bilirubin concentrations, had significantly lower body mass indices and better lipid profiles than the healthy control group, especially at older ages [12]. These findings were supported by animal studies, in which mice with elevated total and indirect bilirubin levels had lower body fat and less hepatic lipid accumulation compared to a control group after a high-fat diet [13]. Furthermore, in mice with diet-induced obesity, the administration of bilirubin reduced the animals’ body weight and total cholesterol concentrations, while their insulin sensitivity increased [14]. Therefore, cumulating evidence indicates a preventive effect of bilirubin against weight gain and metabolic disorders.

Despite the seemingly increasing importance of bilirubin in medicine, the literature on physiological bilirubin concentrations in healthy subjects, especially in infants, remains limited. To fill this scientific gap and because we hypothesized associations with different parameters, we established reference values for bilirubin. We investigated the associations between bilirubin levels (total, direct, indirect) and body weight, the concentrations of iron-related parameters as well as sex hormone levels in a cohort of healthy three- and six-month-old infants.

## 2. Materials and Methods

### 2.1. Study Population

Data were collected within the prospective and population-based German longitudinal study LIFE Child, which is part of the “Leipzig Research Centre for Civilization Diseases” of the University of Leipzig and investigates the influence of various factors on child development as well as on diseases of civilization, especially obesity. Since 2011, numerous anthropometric, laboratory diagnostic, psychological and socio-demographic data have been collected from healthy children [15,16].

The following analysis included all study participants whose bilirubin levels were determined at least at the third or sixth month of life and who were not taking prescription medications known to affect bilirubin or ferritin levels. As a result, data from 411 visits from 28 June 2011 to 7 January 2015 were included in the statistical analysis. None of the children were suspected of having liver disease, hematological disorder, metabolic illness, endocrine abnormality, chronic inflammatory illness, congenital infection (such as toxoplasmosis or CMV) or dietary deficiencies in vitamin D or iron.

### 2.2. Laboratory Measures

Venous blood samples were taken and gathered in EDTA- and Serum-Monovettes from SARSTEDT AG & Co. KG (Nümbrecht, Germany). The samples were processed by trained staff of the Leipzig Medical Biobank according to standard operating procedures. Infants were not fasting during this study. After blood sampling, the immediate laboratory analysis was conducted in the Institute of Laboratory Medicine, University Hospital of Leipzig. Concentrations of several blood parameters were obtained by different analytical techniques.

Total and direct bilirubin concentrations were determined by photometry (diazo method) using the Cobas 8000 analyzer (c-module; Roche Diagnostics, Mannheim, Germany). Indirect bilirubin levels were calculated by subtracting direct bilirubin levels from total bilirubin levels. Hemoglobin concentrations were measured using the platforms XN-10 and XE2100 (Symex, Norderstedt, Germany).

Steroid hormone concentrations of estrogen, testosterone, progesterone, 17-OH-progesterone and androstenedione were determined using liquid chromatography with tandem mass spectrometry (LC-MS-MS) [17]. The electrochemiluminescence immunoassay method (e-module) was used to determine the ferritin concentrations, and the immunological turbidity test (c-module) was applied to detect the transferrin levels. Both analyses were likewise conducted with a Cobas 8000 analyzer (Roche Diagnostics, Mannheim, Germany).

### 2.3. Anthropometric Data

Current body weight of undressed infants was determined using an infant scale. Their body length was measured without diapers and with bare feet using the baby-measuring device “Dr. Keller II”.

Body weights and heights, which had been collected during previous healthy children clinics (German: Kinder-Vorsorgeuntersuchungen), were taken from the children’s health booklet. In Germany, obligatory healthy children clinics take place at the following times of life: after birth (U1), third to tenth day (U2), fourth to fifth week (U3), third to fourth month (U4), sixth to seventh month (U5), tenth to twelfth month (U6), 21st to 24th month (U7), 34th to 36th month (U7a), 46th to 48th month (U8) and 60th to 64th month (U9) [18].

### 2.4. Statistical Analysis

Data were analyzed using the free statistical software R version 4.1.1 [19].

First, we performed a descriptive analysis of our study population by obtaining the mean and standard deviation. Since not all blood parameters were collected from all children, we additionally reported the number and percentage of included values in the age and sex subgroups.

Body mass index and ponderal index were calculated from measured body weight and height at the time of blood sampling as well as from healthy children clinics’ data.

Differences in bilirubin concentrations between age groups and sexes were determined by Welch’s *t*-test. Percentiles for bilirubin levels at three and six months of age were established as the empirical 2.5th and the 97.5th percentile. The confidence intervals for the 2.5th and the 97.5th percentiles were calculated using the adjusted bootstrap percentile (BCa) method. Subsequently, associations between bilirubin levels and concentrations of various blood as well as anthropometric parameters were investigated and tested for statistical significance using linear regression analysis. We checked for heteroscedasticity, non-linearity, influential points and non-normality of errors by diagnostic plots (scale–location plots, quantile–quantile plot, residual–leverage plot and residuals vs fitted). The considered parameters comprised body weight, body mass index, ponderal index, levels of various sex hormones, hemoglobin, ferritin and transferrin. Because we expected the associations to differ between age groups and between boys and girls, we looked at them stratified by both age and sex. We performed a sensitivity analysis to determine whether the exclusion of preterm infants (<37 weeks gestational age) changed the results. This was not the case. The use of hormonal contraceptives by the mothers had no effect on the infants’ bilirubin concentrations. Associations between weight at healthy children clinics and bilirubin levels at three and six months of age were adjusted for sex. Differences or associations described as significant refer to the statistical significance level α = 0.05. We performed a correction for multiple testing and added adjusted *p*-values (Benjamini–Hochberg procedure).

Our analysis is based on base R function provided within the default package stats. Further, we used tidyverse packages [20] for data wrangling tasks (dplyr, tidyr and purrr). Preparing the results for publication (in a cosmetic sense) we used the broom package [21] and custom self-programmed functions (mainly for unified formatting of numbers). Visualization was performed using ggplot2 [22] supplemented with the scales package [23].

## 3. Results

### 3.1. Characteristics of the Study Population

Bilirubin concentrations were collected from 206 (99 girls) infants at three months of age and 205 (97 girls) children at six months of age. In 100 study participants, bilirubin levels were available at both ages. The following delivery methods were reported: spontaneous vaginal birth (70%), caesarean section (22%), assisted (operative) vaginal birth (6%) and others (2%). Nine (4.4%) three-month-old infants and ten (4.9%) six-month-old infants were born preterm (<37 weeks gestational age). Among the three-month-old infants, 180 (87.4%) were breastfed for at least two months. One hundred and fifty (73.2%) six-month-old infants were breastfed for at least five months. Almost all infants received supplementation with vitamin D.

The basic characteristics of our study population are shown in Table 1. Additional descriptive statistics of our cohort are shown in Tables S1 and S2.

At three and six months of age, the boys were 1.9 cm and 2.6 cm taller (*p* < 0.001) as well as 0.55 kg and 0.81 kg heavier (*p* < 0.001) than the girls. While the boys also achieved approximately 0.5 kg/m^2^ higher body mass indices at three (*p* = 0.004) and six (*p* = 0.004) months of age, their ponderal index did not differ from that of the girls.

The six-month-old infants achieved 6.13 µmol/L lower total, 1.73 µmol/L lower direct and 3.22 µmol/L lower indirect bilirubin levels than the three-month-old infants (*p* < 0.001). Bilirubin concentrations did not differ significantly between the sexes, neither at the age of three months nor at the age of six months.

The boys and girls did not differ significantly in their ferritin, transferrin and hemoglobin concentrations. However, ferritin levels were 195 ng/mL higher (*p* < 0.001) and transferrin levels were 0.3 g/L lower (*p* < 0.001) at three months of age than at six months of age. Hemoglobin levels were 0.7 g/dL higher at six months of age than at the age of three months (*p* < 0.001).

Sex hormone concentrations were available only for the six-month-old infants. The girls had 13.5 pmol/L higher levels of estradiol (*p* < 0.001), 0.06 nmol/L higher levels of 17-hydroxyprogesterone (*p* = 0.014) and 0.16 nmol/L higher levels of androstenedione (*p* < 0.001) than the boys. In contrast, testosterone levels were 0.96 nmol/L higher in the boys than in the girls (*p* < 0.001). We found no statistically significant sex difference in progesterone concentrations.

Descriptive statistics of the anthropometric data of our cohort for each of the healthy children clinics are shown in Table S3.

### 3.2. Reference Values for Bilirubin and Associations with Breastfeeding

**Figure 1:**
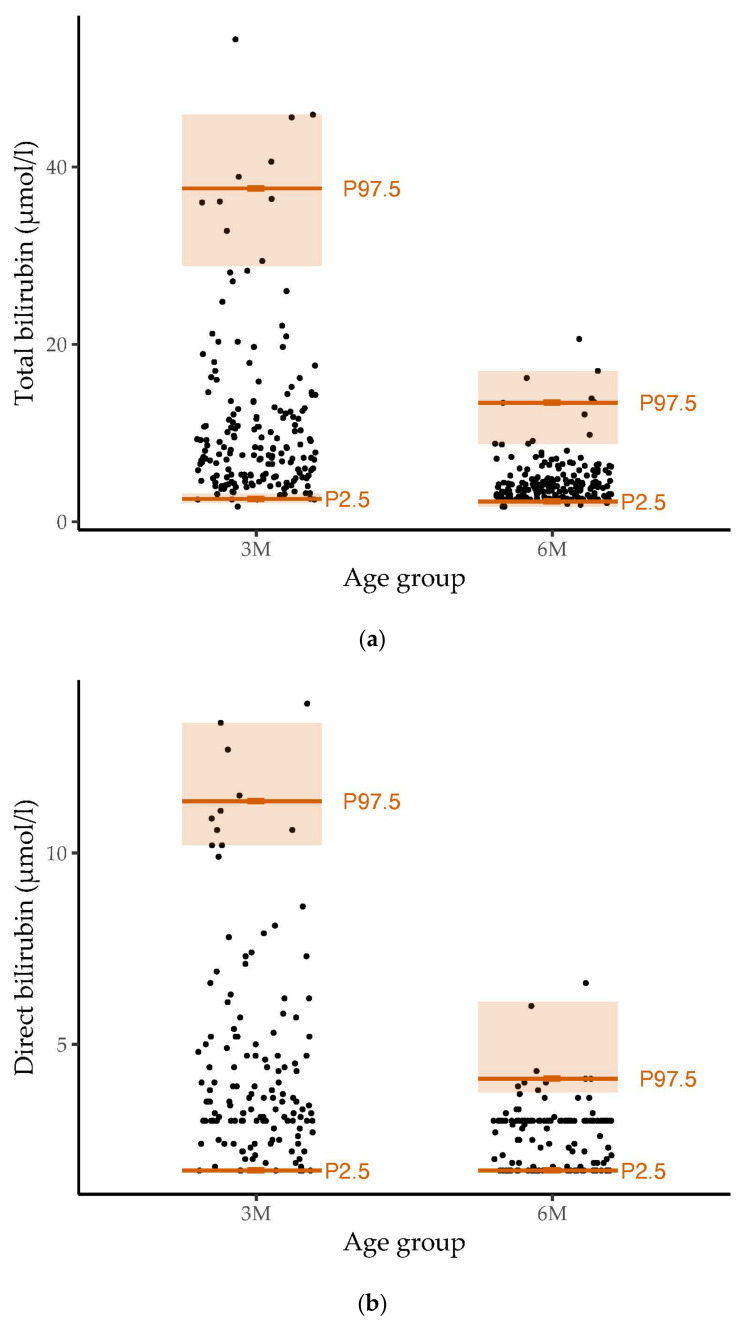

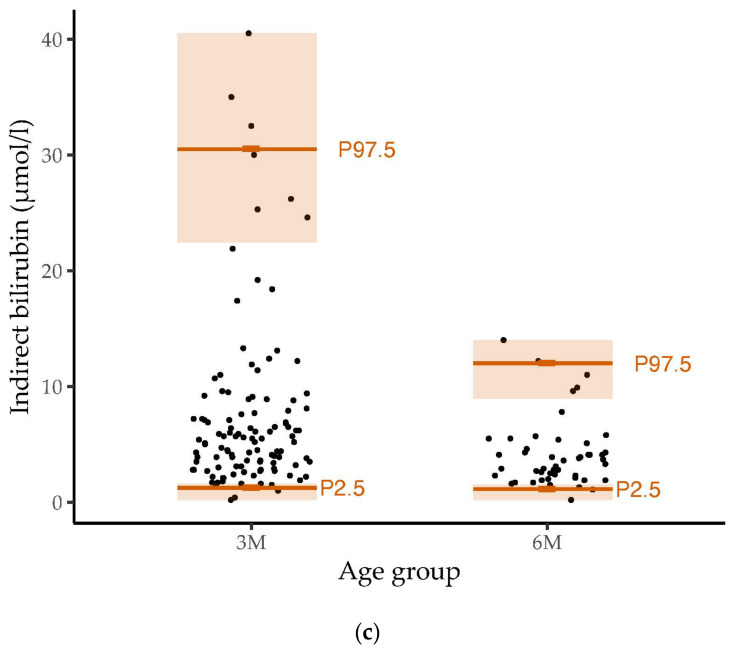
Distribution and reference ranges of (**a**) total (*n* = 376), (**b**) direct (*n* = 290) and (**c**) indirect (*n* = 161) bilirubin values in our healthy study population of infants at the age of three and six months. Dots represent the distribution of measured bilirubin values. The 2.5th percentile (P2.5), 97.5th percentile (P97.5) and the 95% confidence interval are marked in orange The reference ranges for bilirubin at the age of three months were wider than those at six months of age.

The distribution and reference ranges of total, direct and indirect bilirubin values in our cohort are shown in Figure 1.

The 2.5th (with a 95% confidence interval), 10th, 25th, 50th, 75th, 90th and 97.5th (with 95% confidence interval) percentiles for the total, direct and indirect bilirubin levels in the three- and six-month-old infants are listed in Table S4. The percentiles showed decreasing values from three months to six months. At three months, bilirubin values showed greater variability than at six months of age.

At the age of three months, breastfeeding (180 infants) was associated with higher levels of total (*p* < 0.001, *p*adj < 0.001), direct (*p* < 0.001, *p*adj = 0.006) and indirect (*p* = 0.019, *p*adj = 0.325) bilirubin. However, the number of non-breastfed infants was small (26 three-month-old infants). No statistically significant associations were found between breastfeeding and bilirubin levels at six months of age.

### 3.3. Associations between Body Weight and Bilirubin Levels

At three months of age, the association between current weight and total bilirubin levels (*b* = −3.00, 95% CI −4.82 to −1.17, *p* = 0.001, *p*adj = 0.078) or direct bilirubin concentrations (*b* = −1.01, 95% CI −1.64 to −0.38, *p* = 0.002, *p*adj = 0.078), corrected for sex, was statistically significant. Current weight and indirect bilirubin levels were not significantly associated.

The direction of the association between weight and bilirubin levels did not differ between sexes, but the effect size and significance differed between boys and girls. The weight of the three-month-old girls was significantly associated with total (b = −3.41, 95% CI −6.39 to −0.43, *p* = 0.025, *p*adj = 0.325), direct (*b* = −1.32, 95% CI −2.48 to −0.16, *p* = 0.026, *p*adj = 0.325) and indirect bilirubin levels (*b* = −3.71, 95% CI −7.29 to −0.12, *p* = 0.043, *p*adj = 0.416) measured on the same day. Among the three-month-old boys, this association was only statistically significant for total (*b* = −2.74, 95% CI −5.07 to −0.42, *p* = 0.021, *p*adj = 0.325) and direct bilirubin (*b* = −0.87, 95% CI −1.63 to −0.12, *p* = 0.024, *p*adj = 0.325) levels but not indirect bilirubin concentrations.

**Figure 2:**
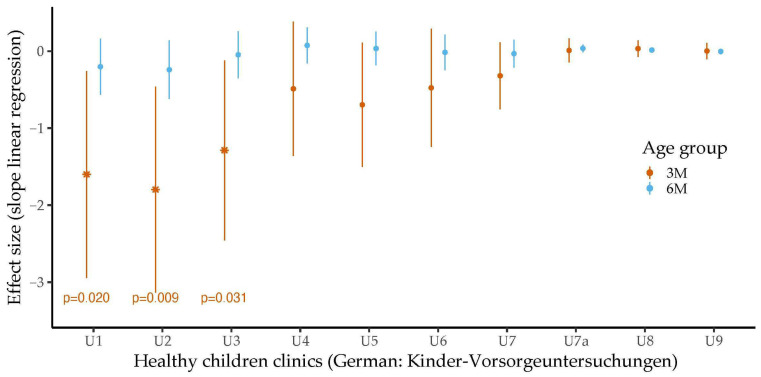
Associations between total bilirubin levels at three and six months of age and body weight during healthy children clinics (German: Kinder-Vorsorgeuntersuchungen) stratified by age and adjusted for sex. The association between bilirubin levels measured at three months of age and body weight is depicted in orange. The association between bilirubin levels measured at six months of age and body weights is depicted in light blue. Effect sizes were determined by linear regression analyses. The error bars show the 95% confidence intervals. Associations with statistical significance are marked. In general, total bilirubin levels were negatively associated with body weight. Statistical significance was reached for the associations between total bilirubin levels at three months of age and weight at healthy children clinics after birth (U1), third to tenth day after birth (U2) and fourth to fifth week after birth (U3).

There were no statistically significant associations between the bilirubin levels at six months of age and the body weights determined on the same day. The relation between total bilirubin concentrations determined at three and six months of age and weight at the healthy children clinics is shown in Figure 2.

Higher body weights at the healthy children clinics were consistently associated with lower total bilirubin values at three months of age. However, only at U1, U2 and U3 did the association reach statistical significance (U1: *b* = −1.60, 95% CI −2.94 to −0.26, *p* = 0.020, *p*adj = 0.325; U2: *b* = −1.80, 95% CI −3.13 to −0.46, *p* = 0.009, *p*adj = 0.193; U3: *b* = −1.29, 95% CI −2.46 to −0.12, *p* = 0.031, *p*adj = 0.357). In addition, a lower weight at U2 was significantly associated with higher concentrations of direct bilirubin, measured in the third month of life (*b* = −0.48, 95% CI −0.95 to −0.01, *p* = 0.047, *p*adj =0.440). Body weight at the healthy children clinics did not show statistically significant associations with levels of indirect bilirubin or bilirubin levels (total, direct or indirect) at six months of age.

Regarding the BMI and the ponderal index, the direction of the associations was predominantly the same, but the results showed no consistent pattern with only occasional statistically significant effects.

### 3.4. Associations between Concentrations of Ferritin/Transferrin and Bilirubin Levels

**Figure 3:**
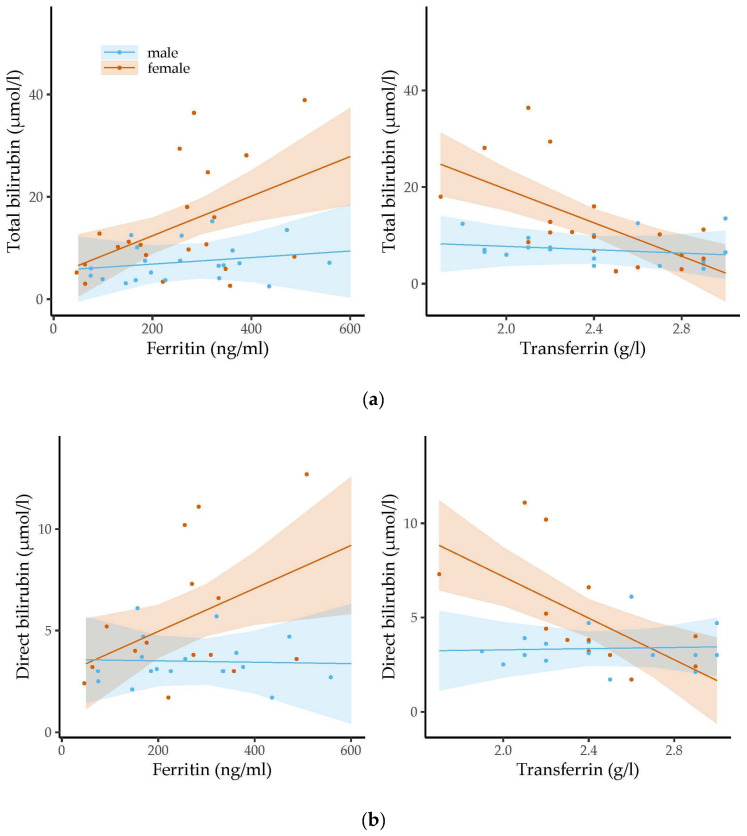

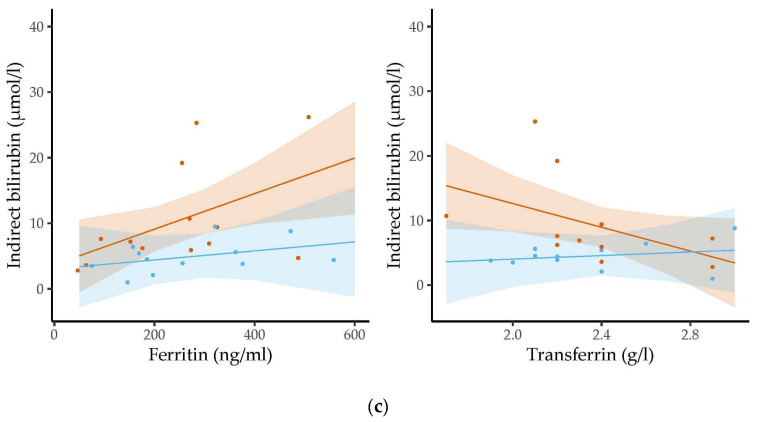
The associations between the levels of ferritin/transferrin and concentrations of (**a**) total bilirubin, (**b**) direct bilirubin and (**c**) indirect bilirubin were assessed stratified by sex in three-month-old infants using linear regression analysis. Dots represent the individual measurements. The semi-transparent background shows the confidence region of the associations. Levels of iron-related parameters were related to bilirubin levels in girls but not in boys. Whereas ferritin levels were positively associated with the levels of the three types of bilirubin, transferrin concentrations were inversely related to bilirubin concentrations.

Figure 3 shows the associations between the levels of the bilirubin molecules and the ferritin or transferrin levels at the age of three months.

Higher ferritin levels were significantly related to higher total (*b* = 0.04, 95% CI 0.01 to 0.06, *p* = 0.004, *p*adj = 0.125), direct (*b* = 0.01, 95% CI 0.00 to 0.02, *p* = 0.025, *p*adj = 0.325) and indirect bilirubin levels (*b* = 0.03, 95% CI 0.00 to 0.05, *p* = 0.022, *p*adj = 0.325) in the three-month-old girls. Higher transferrin concentrations were significantly associated with lower concentrations of total (*b* = −17.29, 95% CI −25.82 to −8.77, *p* < 0.001, *p*adj = 0.024) and direct bilirubin (*b* = −5.52, 95% CI −8.76 to −2.28, *p* = 0.002, *p*adj = 0.078) in the three-month-old girls. We also found a negative trend between transferrin levels and indirect bilirubin levels, but this did not reach statistical significance (*b* = −9.18, 95% CI −18.44 to 0.08, *p* = 0.052, *p*adj = 0.475). In contrast, for the three-month-old boys, we found no association between bilirubin levels and the levels of iron-related parameters. We did not observe any statistically significant relations between the levels of the two iron parameters and bilirubin concentrations in the six-month-old infants.

### 3.5. Associations between Concentrations of Selected Sex Hormones and Bilirubin Levels

Boys with higher testosterone concentrations presented higher concentrations of total, direct and indirect bilirubin. However, only the association with the total bilirubin level reached statistical significance (*b* = 0.68, 95% CI 0.05 to 1.31, *p* = 0.035, *p*adj = 0.357). In girls, we found a positive trend of higher bilirubin levels associated with lower estradiol levels. However, this association was not statistically significant.

Looking at other sex hormone concentrations, statistically significant associations were found especially for indirect bilirubin levels in the girls. Here, higher levels of progesterone (*b* = 26.64, 95% CI 10.74 to 42.53, *p* = 0.002, *p*adj = 0.078) and androstenedione (*b* = 12.16, 95% CI 3.92 to 20.40, *p* = 0.006, *p*adj = 0.144) were associated with higher concentrations of indirect bilirubin. The association between indirect bilirubin levels and 17-hydroxyprogesterone levels did not reach statistical significance (*p* = 0.067, *p*adj = 0.480). In the boys, only the association with progesterone levels was statistically significant (*b* = 22.33, 95% CI 2.00 to 42.67, *p* = 0.033, *p*adj = 0.357). No consistent pattern or statistically significant associations were found between total or direct bilirubin levels and the concentrations of progesterone, 17-hydroxyprogesterone and androstenedione.

## 4. Discussion

### 4.1. Principal Findings

Our study has shown that bilirubin levels are significantly related to body weight, levels of iron metabolism parameters and sex hormone concentrations in the third and sixth months of life. These associations differed by sex and age. Furthermore, the statistical significance and effect size of these associations depended on the type of bilirubin.

### 4.2. Reference Values for Bilirubin and Associations with Breastfeeding

At three months of age, bilirubin levels were higher and the reference ranges for the bilirubin types were wider than at the age of six months. Bilirubin concentrations did not differ between girls and boys. These results are in accordance with previous studies in infants, which also found no differences in bilirubin levels between sexes [24,25] and described an age-related decrease in bilirubin concentrations [24,26].

In most studies, infants of different months of age were grouped to establish reference values for bilirubin [24,25,27]. Compared to a study in healthy three- and six-month-old infants from Helsinki, we found slightly higher 97.5th percentiles for total and direct bilirubin levels at three months of age and lower 97.5th percentiles for bilirubin values at six months of age. However, the 2.5th percentiles for bilirubin were similar [26].

As in our study, breastfeeding was associated with higher bilirubin levels at three months of age [26]. The causes for higher bilirubin levels in breastfed infants are not completely understood. Nevertheless, it has been described that insufficient breastfeeding, including a lower frequency of breastfeeding, leads to a decrease in defecation and consequently reduces bilirubin excretion [28]. In addition, the composition of breast milk and a genetic predisposition appear to influence bilirubin concentrations [29,30].

### 4.3. Associations between Body Weight and Bilirubin Levels

The children with a higher body weight had lower levels of bilirubin. In particular, bilirubin concentrations at the age of three months were significantly related to weight parameters. Moreover, higher total bilirubin concentrations at three months of age were significantly associated with a lower birth weight. Higher bilirubin concentrations in infancy were also associated with lower body weights in later life. However, these associations were not significant. These results are consistent with previous studies that also found an inverse association between bilirubin levels and body weight [31,32,33]. They are also consistent with studies indicating that newborns with higher bilirubin concentrations have lower body mass indices in later life [11].

Another study supports our findings by showing a lower weight shortly after birth in healthy newborns with higher bilirubin levels within the first 48 h of life [32]. Our findings are further supported by studies that found associations between the levels of laboratory parameters of lipid metabolism or metabolic syndrome and bilirubin levels in animals [34], adults [35,36] and obese children [37].

Recent mouse models explain the possible weight-reducing effect of bilirubin. It may improve metabolic function and, thereby, reduce lipid accumulation by binding the peroxisome proliferator-activated receptor-α [10,38]. We observed the strongest effects for the association between weight and total bilirubin levels. Other studies have also reported varying degrees of associations between the levels of the different bilirubin types and the concentrations of various parameters [33,39,40].

### 4.4. Associations between Concentrations of Ferritin/Transferrin and Bilirubin Levels

In three-month-old girls, we found higher ferritin concentrations significantly associated with higher bilirubin levels of all three types, whereas higher transferrin concentrations were significantly associated with lower levels of total and direct bilirubin. After birth, fetal hemoglobin is degraded and replaced by its adult form [41,42]. During hemolysis, in addition to bilirubin, increased amounts of iron are released, which are stored to a certain extent as ferritin. As a negative acute-phase protein and transport protein of iron, transferrin is additionally increasingly loaded with iron [43]. This results in an increase in transferrin saturation, while less unloaded transferrin is measured.

An explanation for the statistically significant associations exclusively in three-month-old infants is that fetal hemoglobin is still being exchanged by adult hemoglobin at this age. In contrast, at six months of age, most fetal hemoglobin has already been substituted [41,42].

While it is known that girls at an older age have lower hemoglobin and ferritin concentrations than boys [44], other studies have shown that, during infancy, boys have significantly lower serum levels of ferritin and hemoglobin than girls [45,46,47].

The exact mechanism of the supposed differences in iron metabolism in girls and boys has yet to be understood. However, current studies describe a gender-specific difference in the activity of the protein hepcidin, which regulates iron metabolism [48,49].

In contrast to these studies, we did not find any sex-specific differences in hemoglobin, ferritin and transferrin concentrations. Nevertheless, statistically significant associations between bilirubin levels and levels of ferritin or transferrin were only observed among girls. We assume other factors may influence the relation between bilirubin levels and the levels of iron parameters. For instance, sex hormones, nutrition and body composition also appear to affect erythropoiesis, iron metabolism and bilirubin concentrations [45,50,51,52,53,54].

In particular, it is important to emphasize that our study population consisted of healthy infants without pathological hemolysis. This could also explain why we found no statistically significant association between the concentrations of hemoglobin and bilirubin levels.

### 4.5. Associations between Concentrations of Selected Sex Hormones and Bilirubin Levels

Higher testosterone levels were significantly associated with higher bilirubin levels in the boys. In the girls, higher concentrations of androstenedione were significantly related to higher levels of indirect bilirubin. Beyond that, higher levels of progesterone were significantly associated with higher levels of indirect bilirubin, irrespective of sex. Previous studies have already reported the influence of sex hormones on bilirubin metabolism [9,55,56,57]. A case report regarding newborns also described an association between the concentrations of progesterone and estradiol and indirect bilirubin levels 72 h after birth [55].

While most previous studies have shown higher bilirubin levels in male than in female children and adolescents due to hormonal changes after the onset of puberty [33,37], the bilirubin concentrations of the girls and boys did not differ in our infant study population. Nevertheless, we have seen that there is already an association between sex hormone levels and concentrations of bilirubin at the age of six months, the time of minipuberty [58].

Although the exact mechanism of the relation between bilirubin levels and sex hormone concentrations is still unknown, animal experiments have provided possible explanations. It has been observed that testosterone suppresses hepatic uridine diphosphate-glucuronosyltransferase activity in rats, while progesterone increases the enzyme activity [9]. Furthermore, the influence of sex hormones on the binding properties of the transport protein albumin and, thus, on bilirubin metabolism is being discussed [56]. Regarding the cortisol precursor androstenedione, cortisol has been reported to be involved in regulating heme oxygenase [57].

### 4.6. Strengths and Limitations

In particular, our study is characterized by a large number of healthy infants and a large body of information. To date, few studies have been carried out in healthy infants, as it is necessary to obtain the consent of their legal guardians for blood sampling, a high number of healthy volunteers and a sufficient volume of blood to establish reference values and to determine a wide variety of laboratory parameters. In contrast to other studies, we considered levels of three bilirubin types and did not exclusively focus on their association with concentrations of testosterone and estradiol but also with levels of other steroid derivates, such as progesterone, 17-hydroxyprogesterone and androstenedione. Moreover, we investigated their relation to body weight at different times of life.

The limitations of our study are the relatively small sample sizes of the subgroups compared to the total. As the infants did not fast before blood withdrawal for ethical reasons, the feeding method and frequency could have influenced the concentrations of the laboratory parameters. Moreover, our study design only allows us to investigate associations. Therefore, we cannot come to conclusions about causal relationships. Other factors such as maternal variables could have influenced bilirubin levels, body weight, levels of ferritin and transferrin, sex hormone concentrations and, therefore, our findings. Therefore, our models have no predictive power. As we have provided reference values for a very homogeneous cohort from Germany, predominantly consisting of subjects of Caucasian ethnicity, our results may not be transferable to more ethnically diverse populations. Moreover, our results are limited in their applicability to the general population, as the participants in the LIFE Child study tend to have a higher socioeconomic status [15], which is associated with better health in general [59].

To date, there are no studies on a cohort of healthy children of a similar age. Moreover, there needs to be more detailed follow-up data on bilirubin levels in the first year of life and thereafter. Therefore, additional studies are needed to exploit the preventive and therapeutic potential of bilirubin.

## 5. Conclusions

The present study established reference values for bilirubin at the age of three and six months in a cohort of healthy infants from Germany. Further, statistically significant relations were shown between the levels of bilirubin types and body weight, levels of iron parameters as well as concentrations of several steroid hormones. Therefore, our results are an important contribution to address the lack of studies on bilirubin levels in a large and healthy study population of three- and six-month-old infants. Our reference values may be used in German hospitals and laboratories. Furthermore, our results suggest that, in addition to pathological causes, physiological parameters such as body weight and sex hormone concentrations are associated with bilirubin concentrations and should be taken into consideration during the interpretation of elevated bilirubin levels.

## Figures and Tables

**Table 1:** Descriptive statistics of the study population consisting of healthy infants aged three and six months.

	3 Months			6 Months		
	Female	Male		Female	Male	
	*N* = 99	*N* = 107		*N* = 97	*N* = 108	
	Mean (±SD)	Mean (±SD)	*p* Value	Mean (±SD)	Mean (±SD)	*p* Value
Age (years)	0.24 (0.04)	0.23 (0.04)	0.405	0.49 (0.04)	0.49 (0.05)	0.772
Height (cm)	59.6 (2.48)	61.5 (2.84)	**<0.001**	66.2 (2.87)	68.8 (2.53)	**<0.001**
Weight (kg)	5.50 (0.60)	6.05 (0.75)	**<0.001**	7.07 (0.87)	7.88 (0.86)	**<0.001**
BMI (kg/m^2^)	15.5 (1.08)	15.9 (1.32)	**0.004**	16.1 (1.31)	16.6 (1.18)	**0.004**
PI (kg/m^3^)	26.0 (2.12)	26.0 (2.43)	0.903	24.4 (2.18)	24.2 (1.79)	0.508
Total bilirubin (µmol/l) *n*	10.7 (7.82) 89 (89.9%)	10.6 (9.98) 93 (86.9%)	0.964	4.62 (3.08) 89 (91.8%)	4.34 (2.30) 105 (97.2%)	0.478
Direct bilirubin (µmol/l) *n*	4.35 (2.47) 60 (60.6%)	4.37 (2.73) 76 (71.0%)	0.969	2.66 (0.84) 69 (71.1%)	2.61 (0.77) 85 (78.7%)	0.680
Indirect bilirubin (µmol/l) *n*	7.16 (5.77) 50 (50.5%)	7.47 (8.29) 63 (58.9%)	0.811	4.74 (3.43) 20 (20.6%)	3.67 (2.50) 28 (25.9%)	0.242
Hemoglobin (g/dl) *n*	11.0 (1.12) 91 (91.9%)	11.0 (0.95) 101 (94.4%)	0.984	11.7 (1.06) 90 (92.8%)	11.7 (1.17) 104 (96.3%)	1.00
Ferritin (ng/mL) *n*	239 (134) 23 (23.2%)	272 (129) 24 (22.2%)	0.397	57.4 (37.3) 24 (24.7%)	63.3 (44.0) 38 (35.2%)	0.573
Transferrin (g/l) *n*	2.39 (0.34) 20 (20.2%)	2.41 (0.37) 22 (20.6%)	0.830	2.68 (0.47) 24 (24.7%)	2.72 (0.37) 38 (35.2%)	0.707
Testosterone (nmol/l) *n*				0.08 (0.06) 34 (35.1%)	1.04 (1.13) 50 (46.3%)	**<0.001**
Estradiol (pmol/l) *n*				51.6 (12.6) 34 (35.1%)	38.1 (3.38) 50 (46.3%)	**<0.001**
Progesterone (nmol/l) *n*				0.34 (0.21) 34 (35.1%)	0.28 (0.07) 50 (46.3%)	0.089
17-OH-progesterone (nmol/l) *n*				0.90 (0.51) 34 (35.1%)	0.65 (0.32) 50 (46.3%)	**0.014**
Androstenedione (nmol/l) *n*				0.39 (0.22) 34 (35.1%)	0.23 (0.15) 50 (46.3%)	**<0.001**

The table shows the mean and standard deviation of the anthropometric and laboratory values in our cohort (for boys and girls at three and six months). Differences in values between sexes were determined using Welch’s *t*-test and are presented as *p*-values. The number of measurements is given for all laboratory values, as not all laboratory values were measured in all subjects. *N* = number of infants in subgroup; SD = standard deviation; *n* = number (percentage) of measurements; BMI = body mass index; PI = ponderal index; 17-OH-progesterone = 17-Hydroxyprogesterone. Statistically significant differences between sexes are marked.

## Data Availability

The datasets generated and/or analyzed during the current study are not publicly available due to ethical restrictions. The LIFE Child study is a study collecting potentially sensitive information. Publishing data sets is not covered by the informed consent provided by the study participants. Furthermore, the data protection concept of LIFE requests that all (external as well as internal) researchers interested in accessing data sign a project agreement. Researchers that are interested in accessing and analyzing data collected in the LIFE Child study may contact the data use and access committee (dm@life.uni-leipzig.de).

## Supplementary Materials

The following supporting information can be downloaded at: https://www.mdpi.com/article/10.3390/metabo14070393/s1. Table S1: Descriptive statistics of the healthy three-month-old infants in our cohort; Table S2: Descriptive statistics of the healthy six-month-old in our cohort; Table S3: Descriptive statistics of the anthropometric data of our healthy cohort during healthy children clinics U1-U9; Table S4: Selected percentiles for total, direct and indirect bilirubin (in µmol/L) of healthy infants at the age of three and six months.
