# Supplementary material for: Bilirubin Levels in Infancy and Their Associations with Body Weight, Levels of Iron-Related Parameters and Steroid Hormone Levels
Source: Metabolites. 2024 Jul 19;14(7):393. doi: 10.3390/metabo14070393 (PMC11279372; doi:10.3390/metabo14070393)
Supplement: Supplementary file 1 [file metabolites-14-00393-s001.zip › Supplementary Material.pdf]

Table S1. Descriptive statistics of the healthy three-month-old infants in our cohort.

	3 Months							
	**Female** *N* = 99				**Male** *N* = 107			
	Mean (±SD)	Median (25% – 75%)	Min – Max	Unknown	Mean (±SD)	Median (25% – 75%)	Min – Max	Unknown
Age (years)	0.24 (0.04)	0.24 (0.21-0.27)	0.14-0.35	0	0.23 (0.04)	0.23 (0.20-0.26)	0.15-0.33	0
Height (cm)	59.6 (2.48)	59.4 (58.1-60.9)	53.4-67.2	0	61.5 (2.84)	61.6 (59.2-63.6)	55.4-68.6	0
Weight (kg)	5.50 (0.60)	5.54 (5.11-5.82)	4.15-7.75	1	6.05 (0.75)	6.02 (5.46-6.63)	4.50-7.83	0
BMI (kg/m^2)	15.5 (1.08)	15.42 (14.65-16.30)	13.21-17.73	1	15.9 (1.32)	15.92 (15.12-16.78)	12.09-19.55	0
PI (kg/m^3)	26.0 (2.12)	26.25 (24.50-27.46)	22.01-30.70	1	26.0 (2.43)	25.64 (24.70-27.43)	18.81-33.31	0
Total bilirubin (µmol/l)	10.7 (7.82)	8.4 (5.2-12.7)	2.6-38.9	10	10.6 (9.98)	7.1 (4.9-12.2)	1.7-54.4	14
Direct bilirubin (µmol/l)	4.35 (2.47)	3.60 (3.00-4.85)	1.70-12.70	39	4.37 (2.73)	3.30 (2.58-5.23)	1.70-13.90	31
Indirect bilirubin (µmol/l)	7.16 (5.77)	5.8 (3.7-7.5)	0.2-26.2	49	7.47 (8.29)	4.5 (2.8-8.5)	0.4-40.5	44
Hemoglobin (g/dl)	11.0 (1.12)	11.00 (10.30-11.80)	8.40-15.00	8	11.0 (0.95)	11.00 (10.50-11.40)	6.61-13.90	6
Ferritin (ng/ml)	239 (134)	255 (141-319)	24-508	76	272 (129)	258 (168-349)	75-558	83
Transferrin (g/l)	2.39 (0.34)	2.40 (2.18-2.63)	1.70-2.90	79	2.41 (0.37)	2.40 (2.13-2.68)	1.80-3.00	85

The table shows the mean, standard deviation, median, interquartile range, minimum, maximum and number of missed values of the anthropometric and laboratory values. *N* = number of children in group; SD = standard deviation, 25%-75% = interquartile range, unknown = number of missed values, BMI = body mass index; PI = ponderal index.

Table S2. Descriptive statistics of the healthy six-month-old infants in our cohort.

	6 Months							
	**Female** *N = 97*				**Male** *N = 108*			
	Mean (±SD)	Median (25% – 75%)	Min – Max	Unknown	Mean (±SD)	Median (25% – 75%)	Min – Max	Unknown
Age (years)	0.49 (0.04)	0.49 (0.46-0.51)	0.39-0.63	0	0.49 (0.05)	0.48 (0.45-0.51)	0.39-0.74	0
Height (cm)	66.2 (2.87)	66.0 (64.8-67.9)	59.2-75.8	2	68.8 (2.53)	69.0 (67.3-70.5)	60.9-75.5	1
Weight (kg)	7.07 (0.87)	7.10 (6.47-7.64)	4.98-9.54	2	7.88 (0.86)	7.87 (7.28-8.47)	6.01-9.73	1
BMI (kg/m^2)	16.1 (1.31)	16.26 (15.7-16.92)	12.25-18.84	2	16.6 (1.18)	16.54 (15.64-17.27)	14.52-19.30	1
PI (kg/m^3)	24.4 (2.18)	24.25 (22.98-25.75)	18.70-30.14	2	24.2 (1.79)	24.10 (22.74-25.57)	19.94-28.22	1
Total bilirubin (µmol/l)	4.62 (3.08)	3.7 (2.6-5.3)	1.7-20.6	8	4.34 (2.30)	3.7 (2.9-4.8)	1.7-17.0	3
Direct bilirubin (µmol/l)	2.66 (0.84)	3.00 (1.80-3.00)	1.70-6.60	28	2.61 (0.77)	3.00 (1.70-3.00)	1.70-6.00	23
Indirect bilirubin (µmol/l)	4.74 (3.43)	3.9 (2.7-5.2)	1.1-14.0	77	3.67 (2.50)	3.0 (1.9-4.4)	0.2-11.0	80
Hemoglobin (g/dl)	11.7 (1.06)	11.80 (11.30-12.28)	7.70-14.00	7	11.7 (1.17)	11.70 (11.10-12.20)	8.20-15.80	4
Ferritin (ng/ml)	57.4 (37.3)	55 (26-76)	3-142	73	63.3 (44.0)	52 (31-94)	9-163	70
Transferrin (g/l)	2.68 (0.47)	2.60 (2.40-2.90)	2.00-4.10	73	2.72 (0.37)	2.77 (2.40-2.98)	2.10-3.60	70
Testosterone (nmol/l)	0.08 (0.06)	0.04 (0.04-0.12)	0.04-0.20	63	1.04 (1.13)	0.59 (0.21-1.50)	0.04-4.31	58
Estradiol (pmol/l)	51.6 (12.6)	52 (37-63)	37-73	63	38.1 (3.38)	37 (37-37)	37-51	58
Progesterone (nmol/l)	0.34 (0.21)	0.30 (0.25-0.35)	0.16-1.37	63	0.28 (0.07)	0.28 (0.23-0.30)	0.16-0.49	58
17-OH-progesterone (nmol/l)	0.90 (0.51)	0.86 (0.50-1.07)	0.30-2.29	63	0.65 (0.32)	0.57 (0.43-0.86)	0.30-1.56	58
Androstenedione (nmol/l)	0.39 (0.22)	0.37 (0.21-0.60)	0.11-0.82	63	0.23 (0.15)	0.20 (0.11-0.31)	0.11-0.66	58

The table shows the mean, standard deviation, median, interquartile range, minimum, maximum and number of missed values of the anthropometric and laboratory values. *N* = number of children in group; SD = standard deviation, 25%-75% = interquartile range, unknown = number of missed values, BMI = body mass index; PI = ponderal index.

Table S3. Descriptive statistics of the anthropometric data of our healthy cohort during healthy children clinics (German: Kinder-Vorsorgeuntersuchungen) U1-U9.

	**Female** *N*= 196									
	**U1**	**U2**	**U3**	**U4**	**U5**	**U6**	**U7**	**U7a**	**U8**	**U9**
n (%)	*194 (99.0)*	*194 (99.0)*	*189 (96.4)*	*190 (96.9)*	*163 (83.2)*	*153 (78.1)*	*133 (67.9)*	*116 (59.2)*	*112 (57.1)*	*89 (45.4)*
Mean height (±SD) in cm	49.4 (2.38)	49.5 (2.41)	53.6 (2.60)	60.4 (2.77)	66.8 (2.90)	73.6 (2.93)	85.7 (3.53)	94.6 (4.05)	103.0 (4.49)	112.0 (5.26)
Mean weight (±SD) in kg	3.36 (0.52)	3.23 (0.52)	4.14 (0.59)	5.76 (0.76)	7.33 (0.83)	8.95 (0.87)	11.92 (1.70)	11.36 (5.98)	12.34 (7.52)	10.77 (9.26)
Mean BMI (±SD) in kg/m^2	13.7 (1.51)	13.1 (1.48)	14.4 (1.29)	15.7 (1.33)	16.4 (1.25)	16.5 (1.30)	16.4 (1.25)	16.1 (1.39)	15.6 (1.38)	15.3 (1.53)
Mean PI (±SD) in kg/m^3	27.7 (3.11)	26.5 (2.99)	26.8 (2.57)	26.0 (2.38)	24.6 (2.20)	22.5 (2.17)	19.0 (2.37)	13.6 (7.14)	11.3 (6.82)	8.07 (6.90)
	**Male** *N*= 215									
	**U1**	**U2**	**U3**	**U4**	**U5**	**U6**	**U7**	**U7a**	**U8**	**U9**
n (%)	*214 (99.5)*	*213 (99.1)*	*212 (98.6)*	*208 (96.7)*	*196 (91.2)*	*185 (86.0)*	*170 (79.1)*	*151 (70.2)*	*144 (67.0)*	*118 (54.9)*
Mean height (±SD) in cm	50.8 (2.23)	50.8 (2.19)	54.9 (2.36)	62.8 (2.95)	68.9 (2.93)	75.8 (3.10)	87.5 (3.88)	96.4 (4.22)	104.0 (4.63)	112.0 (5.52)
Mean weight (±SD) in kg	3.54 (0.51)	3.38 (0.49)	4.51 (0.62)	6.44 (0.82)	8.04 (1.01)	9.75 (1.08)	12.57 (1.64)	12.68 (5.68)	10.48 (8.60)	8.74 (9.93)
Mean BMI (±SD) in kg/m^2	13.7 (1.37)	13.1 (1.30)	14.9 (1.33)	16.3 (1.32)	16.9 (1.46)	16.9 (1.40)	16.5 (1.24)	16.0 (1.59)	15.7 (1.23)	15.5 (1.48)
Mean PI (±SD) in kg/m^3	27.0 (2.70)	25.7 (2.51)	27.1 (2.43)	26.0 (2.41)	24.6 (2.28)	22.4 (2.15)	18.8 (2.26)	14.1 (6.30)	9.27 (7.48)	6.25 (6.98)

N = number of children in group; *n* = number (percentage) of measurements; BMI = body mass index; PI = ponderal index. Times of healthy children clinics: after birth (U1), third to tenth day (U2), fourth to fifth week (U3), third to fourth month (U4), sixth to seventh month (U5), tenth to twelfth month (U6), 21st to 24th month (U7), 34th to 36th month (U7a), 46th to 48th month (U8), 60th to 64th month (U9).

Table S4. Selected percentiles for total, direct and indirect bilirubin (in µmol/l) of healthy infants at the age of three and six months.

Total bilirubin (µmol/l)								
**Age (months)**	***n***	**2.5th (95% CI)**	**10th**	**25th**	**50th**	**75th**	**90th**	**97.5th (95% CI)**
3	182	2.6 (2.12-3.15)	3.9	5.0	7.7	12.4	20.3	37.6 (28.8-45.9)
6	194	2.3 (1.7-2.48)	2.5	2.8	3.7	5.0	7.1	13.4 (8.8-17)
**Direct bilirubin (µmol/l)**								
**Age (months)**	***n***	**2.5th (95% CI)**	**10th**	**25th**	**50th**	**75th**	**90th**	**97.5th (95% CI)**
3	136	1.7 (1.69-1.74)	2.0	3.0	3.5	5.1	7.9	11.4 (10.2-13.4)
6	154	1.7 (1.69-1.70)	1.7	1.7	3.0	3.0	3.2	4.1 (3.74-6.11)
**Indirect bilirubin (µmol/l)**								
**Age (months)**	***n***	**2.5th (95% CI)**	**10th**	**25th**	**50th**	**75th**	**90th**	**97.5th (95% CI)**
3	113	1.2 (0.2-1.6)	2.1	3.1	5.4	7.9	13.3	30.5 (22.4-40.5)
6	48	1.1 (0.2-1.5)	1.7	2.3	3.5	4.7	8.3	12.0 (8.93-14)

n = number of measured values, 90% CI = 90% confidence interval.
